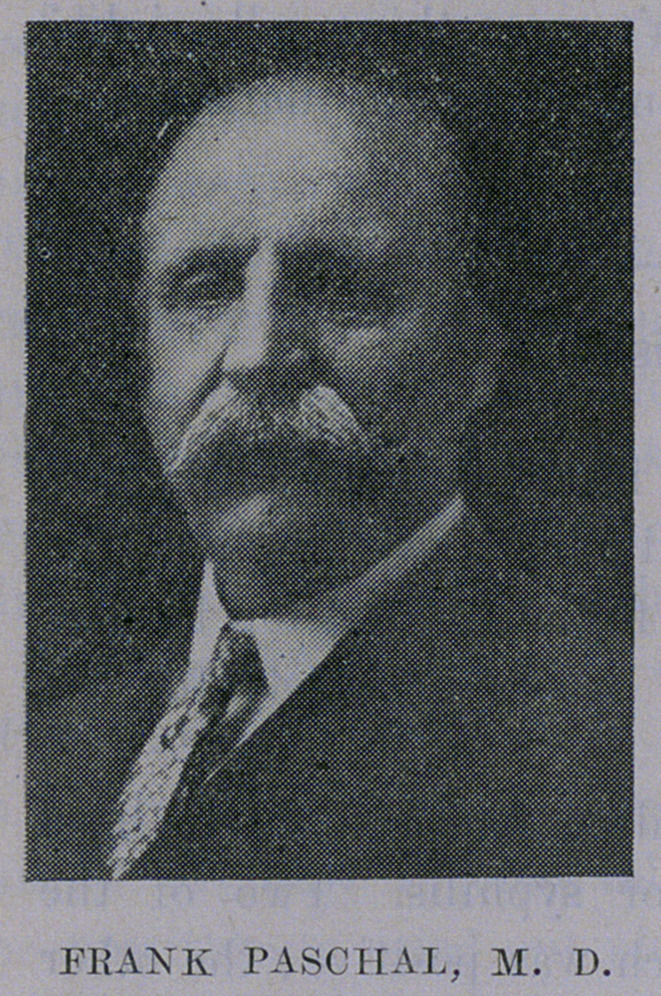# The Necessity for Centralization of Power for Protecting Public Health

**Published:** 1914-07

**Authors:** Frank Paschal


### EDITORIAL DEPARTMENT.

**DR. R. H. L. BIBB, Associate Editor**

DEPARTMENTS { EUGENICS: Drs. M. Duggan and T. Y. Hull.

WOMAN'S DEPARTMENT: Mrs. F. E. Daniel.

### The Necessity for Centralization of Power for Protecting Public Health.

Some of the marvelous results of this century are those obtained by the United States army at home and abroad in correcting unsanitary conditions. Only a few of the many things accomplished can be given, as it would consume too much time and space to attempt going into detail. When Cuba was taken by the United States, yellow fever ceased to be the dread of this country, and the death knell to quarantine—relic of the dark ages—was sounded against preventable diseases. Cholera was stamped out in the Philippines; hookworm eradicated in Porto Rico. Panama, formerly the "graveyard" of the Western Hemisphere, is now a health resort, and the construction of the Canal made possible.

FRANK PASCHAL, M. D.

The occupation of Vera Cruz by the United States army adds another triumph to the many that have gone before. It will be remembered that the first important measure instituted there was the abandonment of San Juan de Ulloa, the foulest and most unsanitary prison on this continent. Prisoners were removed from dark, wet, foul dungeon cells, where they had been treated like brutes. From such treatment many had been driven insane, and were placed in sunlit rooms. This prison had existed for centuries—the Bastille of Mexico—a blot upon civilization. Prisoners held in San Juan de Ulloa, for political reasons, were set free, and others are being humanely cared for. Next followed the cleaning and sanitation of the town. We are told that under Mexican regime that the streets were black with buzzards—nature's scavengers, living on decayed animal matter; that when it accumulated in too great quantities to be eaten by buzzards and it became necessary to get rid of the surplus that buzzards could not cope with, orders would be given to get thirty men, imprisoned for "down and drunk," and put them to sweeping and carting away garbage. Today five hundred men are employed in cleaning the town, and are paid wages. Buzzards no longer infest the streets or perch on housetops. The garbage pile, an accumulation of centuries, is being burned; private houses are inspected; hotels, kitchens and restaurants are forced to be clean and sanitary. In fact, intelligent measures are daily employed to protect public health.

In studying how so much can be accomplished, one is struck by the fact that it is only by the centralization of power. This is the only way that public health can be fully protected. However much one may object to an infringement on State's rights, it is a fact, nevertheless, that until some power, such as that wielded by the Federal government, is employed can full measure of justice in protecting the public health be made possible. State and municipal governments can never measure up to the full standard of what they should be in sanitary matters so long as political appointments for personal or political favors are employed in selecting guardians for public health. It always has and always will continue to suffer when such methods are employed. All matters of public health should be under Federal control. If it were so, it would not be long before tuberculosis would be stamped out, and the death rate from other diseases greatly decreased. Note results in protecting against typhoid fever: "The death rate from typhoid fever per hundred thousand population in the registration area of the United States during the year 1912 (the last report available) was 16.5. The absence of a single death from typhoid in the nearly 100,000 officers and men of the army who were protected by vaccination contrasts favorably with the 16.5 deaths per an equal number of the general population."—C. C. Bass, M. D., in *Am. Journal Trop. Dis. and Prev. Med.*

Perhaps advocating turning over State and municipal health departments to Federal control may not sound well in the ears of politicians, but lives must not be measured by the standard of politics. There is a higher and nobler aim than living for self and selfish purposes. The day may come when there will be an awakening and a realization that centralization of power must be employed in preventing unnecessary loss of life, and until this power is employed, then, and not until then, will the brilliant results attained by the United States army be equaled in protecting public health and humanity.

## Figures and Tables

**Figure f1:**